# Celebrating discovery across the tree of life

**DOI:** 10.1093/g3journal/jkac318

**Published:** 2023-01-12

**Authors:** Lauren M McIntyre

**Affiliations:** Editor in Chief

I am honored to have been appointed as the next Editor in Chief of G3: Genes|Genomes|Genetics. This is an exciting opportunity to continue in the revolutionary community undertaking that is G3. Brenda Andrews, the Inaugural Editor in Chief, is an inspiring leader and she launched G3 with this statement: “Because your research is important to you, it's important to us.” (Andrews *et al*. 2011).

Brenda developed G3 to occupy a unique space in publishing genetics and genomics research, imbuing the journal with an emphasis on rigor in the execution of science and the importance of useful reproducible science in Genes/Genetics/Genomes. I am proud to carry this vision forward. In the nearly 12 years of its existence, we have consistently focused on the needs of our community for an outlet to publish their high-quality research advances, without subjective judgment about the potential impact of the work, and with crisp decisions that do not leave authors wondering about the appropriate next steps. By contextualizing reviews and offering concrete guidance, our peer-editors who are part of your community strive to enhance your work.

G3's peer review and publishing goals embrace science and scientists across the world. Since our 2011 launch we have published work from authors in more than 50 countries on 6 continents. G3 provides a space for our global community to tackle important topics and discuss their findings. Some results are straightforward to interpret; other work may need more space to describe and present important findings. Investigations have a flexible format without strict minimum and maximum page limits—this empowers you to decide how much space is needed for effective communication of your work.

The report format in G3 enables the hard work and time spent in developing resources to receive full credit. Currently, we publish genome reports, mutant screen reports, and software and data resources and we expect to expand the categories that use the report format in future. We publish these reports based on whether the science is useful without injecting narrow opinions of what is interesting—we want you to remember that if it is interesting to *you, it is* interesting.

Useful science is defined by rigorous experimentation, clarity of methods, reproducibility, and data sharing. Data formats that promote data reuse are one way to increase your impact. The development of data standards, and supplementary metadata designed to promote effective and efficient reuse is a key challenge facing our community today. We are committed to meeting this challenge through enhanced supplementary materials that are computer readable and the publication of useful computational tools that extract and integrate information.

**Fig. 1.**
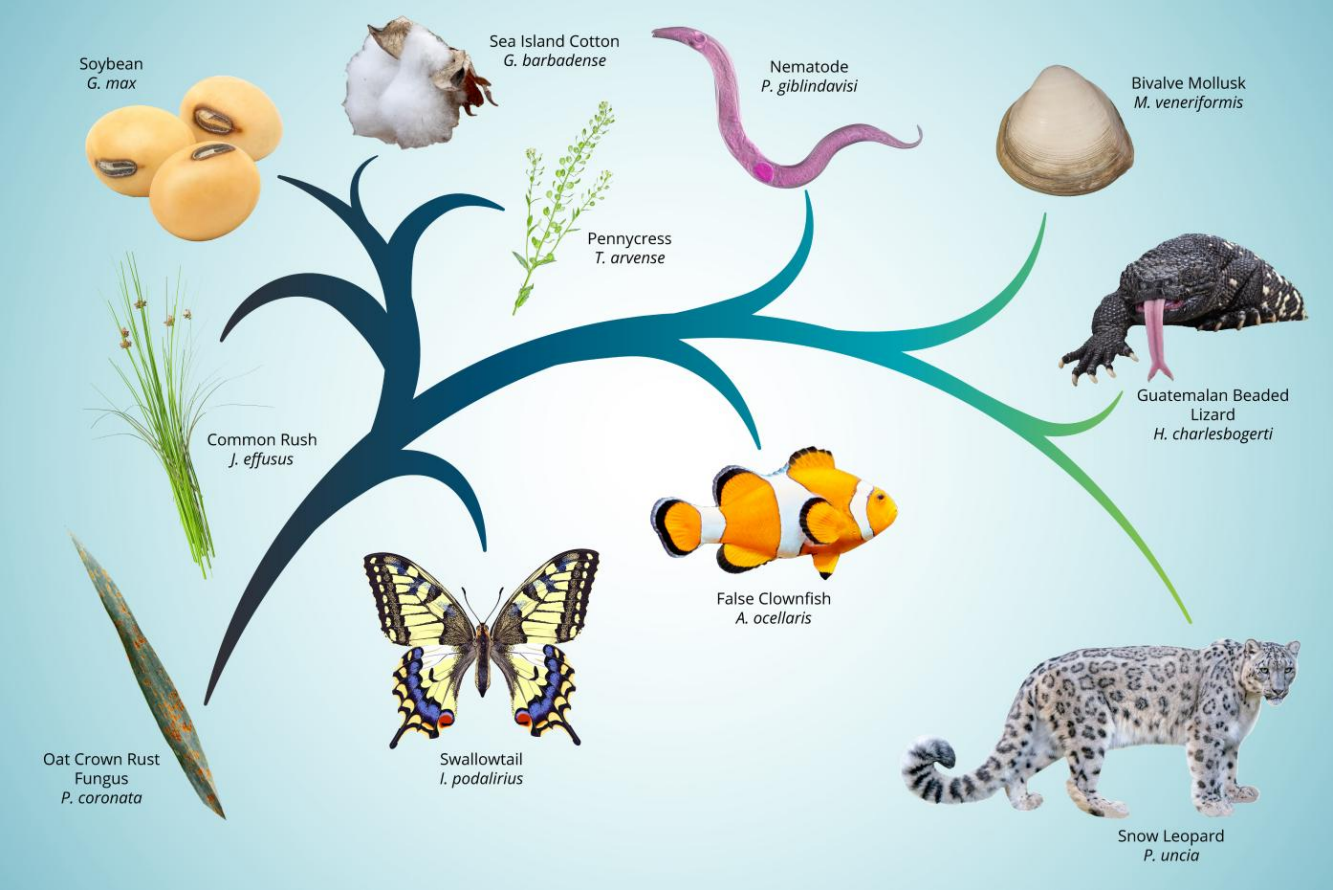
G3 welcomes genome science across the tree of life, life in its variety is fascinating.

Modern technology is empowering our community to ask interesting questions about genes and genomes across the tree of life (Fig. 1) from snow leopards (Armstrong *et al*. 2022), beaded lizards (Dyson *et al*. 2022), False Clownfish (Ryu et al. 2022) and the Bivalve Mollusk (Sun et al. 2022) to and swallowtails (Mackintosh *et al*. 2022) to nematodes (Röseler et al. 2022), pennycress (Navarrete et al. 2022), Sea Island Cotton (Duan et al. 2022), soybean (Yi *et al*. 2022), and Common Rush (Planta et al. 2022). At G3 we are committed to publishing work that seeks to understand the wide range of organisms that you find fascinating, and to publish the bioinformatic tools that enable comparative and functional genomics. As part of this adventure, we embrace the full range of modern techniques, from the hard-core molecular to the detailed mathematical. We revel in the diversity of life and the science that accompanies it.

As practicing scientists, we reject the notion that our science is always neatly packageable. If you have a reproducible result that doesn’t quite fit in the current paradigm, we believe that there should be space for sharing that finding and we are committed to publishing those puzzling findings as a way of stimulating scientific discussion. The publication of any one paper is not the last word, but a part of an ongoing scientific discussion and as such there is room to listen and to explore.

G3 has a unique partnership with our sister journal GENETICS. We work closely together and encourage collaborators to think about how to share credit across papers. Rather than shrinking effort from a collaboration into one dense summary with an encyclopedia-like supplement, we encourage you to think about how to present the work in complementary companion papers. There can be as few as two articles, as in stickleback—where Amores *et al*. (2011) and Stacks (Catchen *et al*. 2011) were published as companions across the two journals, or they can be a multi-institutional collaboration, such as in the Mouse Collaborative Cross (Bottomly *et al.* 2012; Broman 2012a, 2012b; Collaborative Cross Consortium 2012; Gong and Zou 2012; Kelada *et al.* 2012; Lenarcic *et al.* 2012; McIntyre and de Koning 2012; Sun *et al.* 2012; Svenson *et al.* 2012; Threadgill and Churchill 2012; Wang *et al.* 2012; Welsh and McMillan 2012; Zhang, Korstanje, *et al.* 2012; Zhang, Zhang, *et al.* 2012; Zhou *et al.* 2012). As sister journals, our editors work together on publishing collaborative work enabling you to highlight the contributions from all the authors. The editors at the two journals also work together on encouraging discussion on important topics through ongoing collections and series.

I look forward to discussions about how to share data effectively, improve the author experience, increase the impact and visibility of your work and to hearing about and publishing new ways of analyzing metadata. I welcome your input and I am excited to help our community get the maximum benefit from the amazing data we are collecting and sharing.

## Conflicts of interest

None declared.
